# The Impact of Weight Cycling on Health and Obesity

**DOI:** 10.3390/metabo14060344

**Published:** 2024-06-19

**Authors:** Huan Wang, Wenbi He, Gaoyuan Yang, Lin Zhu, Xiaoguang Liu

**Affiliations:** 1 Graduate School, Guangzhou Sport University, Guangzhou 510500, China; 105852023100044@stu.gzsport.edu.cn (H.W.); 105852022100037@stu.gzsport.edu.cn (W.H.); 105852022400308@stu.gzsport.edu.cn (G.Y.); 2 Guangdong Provincial Key Laboratory of Physical Activity and Health Promotion, Guangzhou Sport University, Guangzhou 510500, China

**Keywords:** weight cycling, metabolic diseases, chronic inflammation, weight loss strategy

## Abstract

Obesity is a systemic and chronic inflammation, which seriously endangers people’s health. People tend to diet to control weight, and the short-term effect of dieting in losing weight is significant, but the prognosis is limited. With weight loss and recovery occurring frequently, people focus on weight cycling. The effect of weight cycling on a certain tissue of the body also has different conclusions. Therefore, this article systematically reviews the effects of body weight cycling on the body and finds that multiple weight cycling (1) increased fat deposition in central areas, lean mass decreased in weight loss period, and fat mass increased in weight recovery period, which harms body composition and skeletal muscle mass; (2) enhanced the inflammatory response of adipose tissue, macrophages infiltrated into adipose tissue, and increased the production of pro-inflammatory mediators in adipocytes; (3) blood glucose concentration mutation and hyperinsulinemia caused the increase or decrease in pancreatic β-cell population, which makes β-cell fatigue and leads to β-cell failure; (4) resulted in additional burden on the cardiovascular system because of cardiovascular rick escalation. Physical activity combined with calorie restriction can effectively reduce metabolic disease and chronic inflammation, alleviating the adverse effects of weight cycling on the body.

## 1. Introduction

Obesity and overweight have reached pandemic proportions worldwide, with the prevalence of obesity as high as 20% in Europe and already over 30% in some countries, and projections show that the proportion of obesity will increase further in the future [1]. Obesity or overweight is a global subhealth problem associated with a variety of diseases, including diabetes, cardiovascular disease, hepatic steatosis, and chronic inflammation [2]. People of all ages and occupations are under social, media, family, or psychological pressure to lose weight [3]. Dieting is one of the most popular and easiest methods for people to lose weight. So-called dieting control weight refers to “the behavior of eating in a controlled manner in order to reduce, maintain or increase body weight”, including the control of food intake and food types to achieve the purpose of calorie restriction. The prognosis of dieting and weight loss is limited; it is also hard for individuals who diet to maintain weight without rebounding for a long time, and it is hard for people on diets to keep the weight off for a long time without recovery. On the contrary, they tend to fall into the vortex of weight recovery after weight loss and dieting again [4]. The process of weight loss and recovery is called weight cycling (WC) or weight fluctuation [5]. Weight cycling is also known as the ‘yo-yo diet’ or ‘yo-yo effect’, which refers to the periodic decrease or increase in body weight, similar to the up and down movement of a yo-yo [6].

As the number of individuals with obesity increases, the percentage of those who lose weight increases, which leads to a yearly increase in patients with weight cycling as well [4]. Although weight loss has the beneficial effect of improving some chronic diseases, weight cycling is associated with increased risk of many diseases, such as anxiety, depression, type 2 diabetes, hypertension, cancer, and high mortality [3]. The adverse effects of weight cycling on the body even outweigh persistent obesity [6]. Research has shown that weight cycling increases the risk of disease. In particular, weight cycling leads to metabolic abnormalities in adipose tissue, the cardiovascular system, pancreatic tissue, and the immune system. However, there are few reviews to summarize the disadvantages of weight cycling on the body systematically. Therefore, we endeavor to sort out the mechanism of what leads to a variety of chronic diseases and find a suitable way to lose weight for patients with obesity or overweight.

In this paper, we mainly used the literature research method to analyze the articles we have retrieved. For this work, a bibliographic survey was performed in the search engines PubMed, Web of Science, and Google Scholar, supplemented by a manual, in-depth study of articles and their respective bibliographies. The research was fulfilled by acquiring articles randomized in the last 10 years, using the following keywords: weight cycling, weight loss, weight recovery, chronic inflammation, and metabolic disease. After selecting articles based on their relevance to weight cycling, 99 articles were finally included in the references. Of these 99 references, several were not written within the last 10 years, but they were important to the exploration of weight cyclin.

## 2. Effects of Weight Cycling on Tissues and Organs

### 2.1. Effect of Weight Cycling on Adipose Tissue

Obesity increases the risk of diseases such as diabetes, cardiovascular disease, multiple cancers, arthritis, and periodontitis, but weight loss can greatly reduce the risk of these diseases [7]. However, patients with weight cycling not only fail to maintain long-term weight loss but also have more free fat mass than the original level [6,8,9]. In an experiment involving 125 adults with obesity or overweight, it was found that multiple weight cycling was associated with fat deposition, especially in the central area including the abdomen and waist [10]. After experiencing weight cycling, the skeletal muscle mass (SMM) assessed by magnetic resonance imaging decreased, with the more times of weight cycling, the more significant the ratio of fat mass loss to cumulative lean mass loss decreased [11,12,13]. The reason for the increase in body weight and fat mass after weight cycling may be found in animal experiments. After three times of weight cycling, the mice that were fed a low-fat diet lost less weight, while the mice that were fed a high-fat diet had a higher degree of fat mass gain. The phenomenon indicates that the reduction of relative energy expenditure in weight cycling mice seems to explain why weight cycling patients tend to regain more weight than their initial weight after dieting, and why those with greater fluctuations in fat mass are more likely to recover weight in the future [14]. Reduced energy expenditure in weight cycling mice may be associated with the loss of brown fat. Charlot et al. [15] identified mechanisms by which weight cycling resists weight loss not only by increasing the accumulation of white adipose tissue, such as visceral and subcutaneous fat mass, but also by potentially decreasing the amount of brown fat to reduce energy expenditure associated with calorie loss. In the study of Dankel et al. [16], the weight and fat mass of weight cycling mice increased at the same time. After quantitative PCR experiments, several clock genes were detected, including Dbp, Tef, and Per. The expression of Per2, Per3 Nr1d2 was down-regulated, while the clock gene played a key role in energy homeostasis [17,18]. However, there were also conflicting experimental results; for middle-aged, high-fat-diet-fed mice, weight cycling did not increase body weight or relative adiposity [19]. Body weight and adiposity gains in weight cycling mice were nonsignificant when compared with high-fat-diet-fed mice that did not undergo weight cycling, and there were also gender difference [19]. The effect of weight cycling on body fat is also related to physical status, and a study of patients with type 2 diabetes found that in overweight and obese patients with type 2 diabetes, weight cycling may result in a relative loss of free fat mass or an increase in fat mass [20].

In addition to affecting body fat distribution, free fatty acids, and fat mass, body weight cycling also enhances the inflammatory response of adipose tissue [21]. Obesity causes macrophage infiltration into adipose tissue, and increased production of pro-inflammatory mediators and inflammatory factors in adipocytes. However, the chronic inflammatory state caused by weight cycling has more significant changes than sustained weight gain [9]. Silva et al. [21] led mice through two or three consecutive weight cyclings, which found that weight cycling was related to body weight development and fat bank remodeling in mice, affecting blood lipids, glucose–insulin homeostasis, and adipokine levels, as well as aggravating the inflammatory response of adipose tissue in mice. Weight cycling induces dysregulation of the production and secretion of adipokines, alters homeostasis, and exacerbates chronic inflammation [21], which is one of the incentives to promote a variety of chronic diseases. The effect of weight cycling on adipose tissue changes body composition, regulates clock gene expression, and results in fat expansion, inducing blood glucose disorder and chronic inflammation.

### 2.2. Effect of Weight Cycling on Cardiovascular Metabolism

Weight cycling has a series of effects on cardiovascular metabolism. Cardiovascular metabolic disorders, including coronary heart disease and stroke, are the most common non-communicable diseases in the world, causing 17.8 million deaths in 2017, and the number of deaths is increasing every year [22]. Body mass index (BMI) is positively correlated with the incidence of cardiovascular metabolic disorders and mortality. According to the World Health Organization (WHO) report, with an increasing BMI by 1 kg/m^2^, the risk of cardiovascular disease in men and women increased by 1.18 and 1.14, respectively [23]. Obesity is highly correlated with the incidence of cardiovascular disease, and weight cycling caused by weight loss promotes the incidence of cardiovascular disease. Weight cycling leads to a significant increase in cardiometabolic risk through the accumulation of visceral fat [24]. Additionally, there is a correlation between weight cycling and cardiovascular metabolic diseases, atherosclerosis, and even mortality [7,25]. Mice experiments by Schofield SE et al. [26] found metabolic dysfunction in male weight cycling mice. Increased adipose tissue deposition is associated with a high incidence of cardiovascular disease, and increased adipose tissue in weight cycling mice may be key to inducing metabolic dysfunction [10,26]. In another experiment with rats fed a free-choice high-fat and high-sucrose diet, it was found that the high-sucrose-diet rats had larger ventricular weights and higher blood pressure, but they did not develop arterial endothelial dysfunction [27]. Research on weight cycling and cardiovascular disease suggests that fluctuations in cardiovascular risk variables (e.g., blood pressure, heart rate, sympathetic nerve activity, blood glucose, lipids, and insulin) may repeatedly exceed normal values during weight regain, thereby placing additional stress on the cardiovascular system [4]. Moreover, there are gender and BMI differences in weight cycling and cardiovascular metabolic diseases in people with obesity or overweight. Cardiac metabolic indexes, for example, fasting blood lipids, insulin sensitivity, and blood pressure with weight cycling are more susceptible in women than men [28]. Hypertriglyceridemia, high-density lipoprotein cholesterol, and hypertension were significantly associated with the variability of weight cycling in men with BMI ≤ 25 kg/m^2^, but not in men with BMI ≥ 25 kg/m^2^ [4,28].

The number of times of weight cycling has a different effect on the cardiovascular system. According to the number of times, it can be further divided into mild weight cycling, moderate weight cycling, and severe weight cycling. Its impact on cardiac metabolic health is inconsistent, but the specific degree of harm remains to be studied [29]. The result may be the body’s adaptation to the changes after each weight loss [28,30]. In female patients with weight cycling, mild weight cycling is associated with a reduced risk of cardiovascular mortality [30]. In addition, Jeong et al. [23]. showed that weight maintenance after weight loss has a significant protective effect on cardiovascular disease compared with weight recovery after weight loss and no weight change. This cardioprotective effect of maintaining weight loss may be mediated by improving cardiac metabolism or liver function, but this benefit gradually vanishes in weight cycling. Therefore, in terms of cardiovascular metabolism, weight maintenance after weight loss can produce many benefits for cardiovascular metabolism [6]. Weight cycling gives rise to fluctuations in cardiovascular risk variables, significantly increasing the risk of cardiac metabolism and causing a series of adverse effects on the cardiovascular system.

### 2.3. Effect of Weight Cycling on Pancreatic Tissue

The pancreas is an important organ to maintain the body’s blood glucose balance; abnormal metabolism of pancreatic tissue increases the risk of diabetes [31]. Studies of the metabolic effects of weight cycling on pancreatic tissue have focused on the effects of weight cycling on type 1 and type 2 diabetes mellitus. Animal experiments and clinical experience have shown that weight cycling may be more harmful than simple persistent obesity in increasing the risk of diabetes and cardiovascular disease [8].

The main feature of patients with type 1 diabetes is the autoimmune destruction of pancreatic β cells [32]. Type 2 diabetes is characterized by hyperglycemia due to insufficient insulin secretion of pancreatic β cells in the case of overnutrition or insulin resistance [33]. The frequency and degree of weight cycling are positively correlated with the increased risk of type 2 diabetes and hypertension [34]. Through the proportional hazards model (Cox) proportional hazard analysis, it was found that different age groups, genders, living habits, and body mass index had different effects on the incidence of diabetes in weight cycling patients [35].

Weight cycling increases the incidence of diabetes by impairing the function of β cells, up-regulating or down-regulating the expression of insulin-secreting genes, and β-cell endoplasmic reticulum stress [36]. In the background of obesity and insulin resistance, blood glucose control is mainly through β-cell compensation and subsequent hyperinsulinemia; weight cycling mice do not show the whole-body insulin action as damaged, but this is rather due to impaired pancreatic β-cell buffering of weight changes, resulting in insufficient insulin secretion [37]. The lack of pancreatic adaptation to weight cycling is the core factor for the deterioration of blood glucose control in mice with weight cycling [38]. Insulin secretion is controlled by the quantity and quality of β cells. During weight cycling, the combined effects of sudden changes in blood glucose concentration and hyperinsulinemia lead to an increase or decrease in pancreatic β-cell populations, which can cause β-cell fatigue and lead to β-cell failure [39]. In addition, since β cells are easily influenced by increased oxidative stress, the rapid changes in nutrient load during the conversion of high-fat and low-fat diets may cause excessive burden on β cells and further accelerate their fatigue [40]. There is also some evidence that hyperinsulinemia accelerates the metabolic clearance of C-peptide, which may lead to a decrease in endogenous C-peptide-based insulin secretion [41]. In addition to the decrease in insulin secretion caused by β-cell fatigue failure, it is also related to the down-regulation of related gene expression. The weight cycling down-regulated some β-cell transcription factors’ expression that are essential for maintaining pancreatic plasticity. Compared with fat mice, the cell cycle-related pathways in the weight cycling pancreatic are up-regulated, and the pathways related to tissue protein localization are down-regulated. The expression of islet β-cell function essential factor Mafa, Ddx1, NKX6.1, is reduced [37]. In conclusion, weight cycling is closely related to the incidence of type 1 and type 2 diabetes. The main mechanism is to increase the risk of diabetes by impairing the insulin secretion function of islet β-cells and changing the expression of insulin-related genes.

## 3. Effect of Weight Cycling on Chronic Inflammation

### 3.1. Effect of Weight Cycling on Macrophages

Chronic inflammation caused by obesity is related to the synthesis and secretion of pro-inflammatory cytokines [17,42]. With the increase in body weight, adipose immune cells are involved in the occurrence of obesity-related diseases. Inflammatory cells transfer and infiltrate into adipose tissue, releasing inflammatory cytokines such as Interleukin-1 beta (IL-1β), tumor necrosis factor α (TNFα), and Interleukin-6 (IL-6) [43,44]. These cytokines promote the decomposition of adipocytes, loss of insulin signaling, and accelerate the development of diabetes [43,44]. Even after long-term weight loss, there are still plenty of macrophages in adipose tissue [45]. Macrophages are the main components of immune cells in adipose tissue and are involved in the regulation of body homeostasis in normal weight and metabolic disorders in obesity. They play an important role in both innate and adaptive immune responses, such as phagocytosis of apoptotic and necrotic cells and cell debris, wound healing, fibrosis, and providing host defense against invading pathogens [45,46]. Previous studies have shown that adipose tissue macrophages (ATMS) have different polarization states, which can be divided into M1-like cell (M1) macrophages and M2-like cell (M2) macrophages. The M1 macrophage phenotype is the pro-inflammatory phenotype. Obese adipose tissue is dominated by M1 macrophages, whereas M2 macrophages exhibit an anti-inflammatory phenotype and are predominantly found in lean adipose tissue [45,46].

Moreover, weight cycling and ATMS interact with each other. In both obesity or overweight patients and mice models, macrophage infiltration in adipose tissue is relatively increased. A changing lifestyle or surgical intervention for weight loss both can reduce the number of macrophages in adipose tissue and alleviate fat inflammation [46]. In the early weight loss stage of caloric restriction marked by increased lipolysis, the infiltration of macrophages in adipose tissue increased, and the number of ATMS decreased significantly only after long-term weight loss. Kosteli et al. [47] found that a shooting up of ATMS in the early stage of weight loss was due to the increase in fatty acid content caused by lipolysis, which promoted the expression of chemokines in adipocytes. What is more, weight cycling further promotes the infiltration of macrophages in adipose tissue. Studies have shown that ATMS are still retained in adipose tissue after weight loss, and inflammation-related genes are up-regulated [48,49,50]. A study found that weight loss could not change the immunological response associated with obesity in male mice, which may be due to the fact that immune cells can remember the obesity status of the body [17]. It is speculated that weight cycling induces the innate immune memory of adipose tissue macrophages [17]. However, other research has shown that weight cycling induced by a high-fat diet does not change the number of ATMs or M1 polarization, but changes the number of T-cell populations [51]. There is no doubt that the hyperinfiltration of macrophages in adipose tissue promotes the development of chronic inflammation, which is accelerated by macrophages during weight cycling [46,51]. However, the exact relationship between weight cycling and macrophages still needs further study and clarification.

### 3.2. Effects of Body Weight Circulation on T Cells

T cells are the cellular components of the adaptive immune system, which can consist of multiple cell subsets, including helper T cells (Th), regulatory T cells (Treg), cytotoxic T cells (Tc) and some inherent T-cell subsets [52]. There are many kinds of T cells in obese adipose tissue. Cytokines secreted by T cells are necessary for macrophage activation [53]. Weight cycling affects the infiltration and functional status of T cells in adipose tissue. According to some research, the number of CD4+ and CD8+ cells in adipose tissue and the expression of various helper T cytokine-related genes (Cd3, Cd4, Ill12, Il12rb, Ccl5) were significantly increased during weight cycling [51]. Zou et al. [54] found that mice with a history of obesity exhibited swelling of the spleen and thymus during weight cycling and a significant increase in T-cell subsets including CD4+ and CD8+ in adipose tissue compared to healthy mice. At the same time, the expression of TCRβ and Treg genes in adipose tissue was significantly up-regulated before and after weight recovery. It is speculated that CD4+ T cells can store the body’s obesity memory, mediate obesity, and promote weight recovery, making it difficult to lose weight, and maintain weight after weight loss, and therefore induce weight cycling [55].

The above data suggest that weight cycling is closely related to the up-regulation of inflammatory factors, which induces pro-inflammatory T-cell response and aggravates weight cycling [21,51]. In addition, the percentage of CD4+ and CD8+ cells in adipose tissue and the expression of Th1 cell-derived factors Ifng and Il-12 genes were positively correlated with impaired glucose tolerance in weight cycling mice [51]. T-cell-mediated inflammation may impair insulin sensitivity in adipose tissue and systemic glucose intolerance during the process of weight cycling [51]. In summary, weight cycling may regulate inflammation and glycemic status by affecting T-cell infiltration and ultimately adversely affect the body. However, few studies have examined the effects of weight cycling on T cells, and the mechanisms need to be further investigated.

### 3.3. Effects of Weight Cycling on Other Immune Cells

During the transition from health to obesity, especially during weight loss, recovery, and cycling, the number and inflammatory state of most immune cells in adipose tissue are altered [56,57,58,59]. At present, the research on the effect of weight cycling on chronic inflammation mainly focuses on macrophages and T cells. However, apart from the infiltration of macrophages and T cells in people with obesity, there are also changes in adipose mast cells, B cells, eosinophils, neutrophils, dendritic cells, and natural killer cells [57]. Unfortunately, there is no independent report on the effect of weight cycling on the above factors, but the state and number of these cells change with fluctuations in body weight.

**Figure 1:**
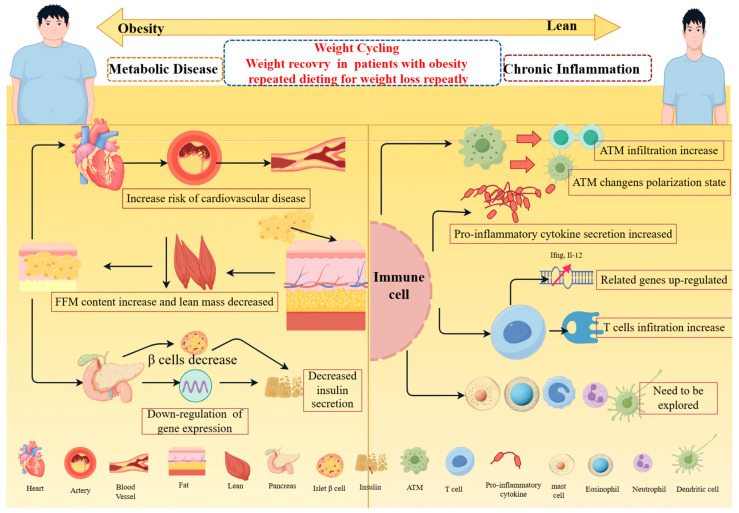
Weight cycling model and associated effects on the human body. Weight cycling is a state of weight change in which individuals lose weight and regain repeatedly. Weight cycling changes body composition, increases visceral and subcutaneous fat accumulation, decreases muscle weight, causes frequent fluctuations of cardiovascular metabolic factors, and increases risk of cardiovascular disease such as coronary heart disease and atherosclerosis by down-regulating the expression of insulin-related genes in islet β cells and damaging the number of islet beta cells resulting in reduced insulin secretion, thus disrupting blood glucose homeostasis. Weight cycling also promotes macrophages’ inflammatory genes’ expression and polarization, increasing the infiltration of macrophages in adipose tissue to exacerbate the chronic inflammatory state of adipose tissue; inducing the transitional pro-inflammatory T-cell response, which may lead to impaired insulin sensitivity and systemic glucose intolerance in adipose tissue during weight cycling. In addition, it has adverse effects on neutrophils, eosinophils, fat mast cells, and β cells. Created using FigDraw (www.figdraw.com, accessed on 20 May 2024).

Adipose mast cells are type 2 immune cells, which have the physiological functions of defending against bacteria and viruses, and have effects on autoimmune and atherosclerosis [60,61]. Adipose mast cells change with weight loss and weight cycling. Body weight homeostasis may affect the lipid treatment and antigen presentation of adipose mast cells. Despite the high prevalence of weight cycling, little research has been conducted on the proportion and function of adipose mast cells in this setting [62]. Future research needs to understand the role of fat mast cells in obesity and weight cycling. B cells are distributed in adipose tissue and participate in the formation of coronary structures related to fat cell death. Obesity accelerates B-cell dysfunction and antibody production. Weight loss caused by caloric restriction or bariatric surgery can reduce B-cell inflammation, lessen systemic B-cell-activated cytokines, and promote B-cell function [63,64,65]. Eosinophils have been reported to affect the homeostasis of adipose tissue by alternately activating macrophages to act on adipocytes, which is related to driving energy consumption. In animal experiments, Wu [66] found that eosinophils led to a significant increase in body free fat content, and also increased fasting blood glucose levels in mice. Regulating the number and functional activity of eosinophils in adipose tissue may be a new direction to cure metabolic disorders, but increasing the number of such cells may cause many side effects related to the pathology of eosinophilia [66,67,68]. Neutrophils are necessary in responding to pathogen invasion. After moderate weight loss in fat patients, neutrophils release E-series resolvins1 (RvE1) significantly, which may determine the function of neutrophils in the case of weight loss [69]. Dendritic cells (DCs) are a type of specific antigen-presenting cells. With the increase in body weight, the activated subsets of dendritic cells increase in obese adipose tissue tend to an activated immune regulatory state. The variation aggravates in weight loss and body weight cycling persistently [56,70,71]. Even so, the role of B cells, eosinophils, neutrophils, and dendritic cells in weight cycling has not been reported. Obesity can lead to chronic inflammation, disrupting the body’s immune homeostasis. Although weight loss can partially treat chronic inflammation, weight cycling may further aggravate the body’s chronic inflammation. In summary, weight cycling increases the incidence of metabolic diseases and induces immune cell infiltration to aggravate the body’s inflammatory response (Figure 1).

## 4. Weight Cycle Model

Weight cycling is a research hotspot in the field of obesity, and the existence of its model provides convenience for exploring the mechanism of weight cycling. At present, the models of weight cycling are mainly divided into human experiments and animal experiments. Among them, human experiments are mainly based on observational studies, while animal experiments are mainly based on mechanism exploration. The onset of weight cycling is accompanied by an increased incidence of many diseases, and we describe the process of weight cycling in humans in order to reveal the underlying pattern of this model formation and thereby further explain animal models of cycling.

The study of weight cycling can be traced back to 1950. The proportion of male and female dieters in the United States has been increasing. After weight loss designedly, about 20–30% of adults said they had a significant weight loss effect. However, about one-third of the population recovers some or all of their lost weight after weight loss, which makes many fat or normal-weight people cycle between weight loss and weight recovery in their lifetime [4,28,72].

A study of weight cycling in people revealed a loss of mobility, low quality of life, and depression. The 72-month weight changes of 731 patients with knee arthritis were included in the analysis. According to the variation during this period, the participants were divided into the weight-stable-thin group (BMI < 25 kg/m^2^), a weight-stable-overweight group (BMI = 25–29.9 kg/m^2^), a weight-stable-obese group (BMI ≧ 30 kg/m^2^), continuous weight loss group, continuous weight gain group, weight gain-loss-gain group and weight loss-increase-loss group [73]. The results showed that the stable weight loss group showed the most severe pain state and the worst physical activity, compared with two weight cycling patterns or one-way weight loss [73]. In order to determine the effect of weight cycling times on human muscle mass and strength, ROSSI et al. [74] divided 60 men and 147 women with obesity (average BMI ≧ 37.9 kg/m^2^) into a non-weight cycling group (cycle times ≧ 1), moderate weight cycling group (cycle times 2–5), and severe weight cycling group (cycle times ≧ 6), according to the number of weight cycles in 2 years. The study found that compared with those without weight cycling or mild cycling, severe weight cycling showed lower ASM/BMI value and grip strength, even assuming the probability of sarcopenia was nearly 6 times higher than in other groups. The ratio between fat mass and lean body mass loss decreased in each weight cycling, and the ratio was positively correlated with weight cycling times [74]. The human body weight cycling model mainly records the weight changes of people with obesity over a long period of time (≧2 years), then according to weight changes divided into several groups, but researchers did not intervene with participants in daily life. This method can restore the weight cycling status of obese or overweight individuals to the maximum extent. However, there are too many uncertainties including diseases, drugs, and exercise during the experiment. The range of span and weight in weight cycling is not clearly defined, which affects the accuracy of the experimental results to a certain extent.

**Figure 2:**
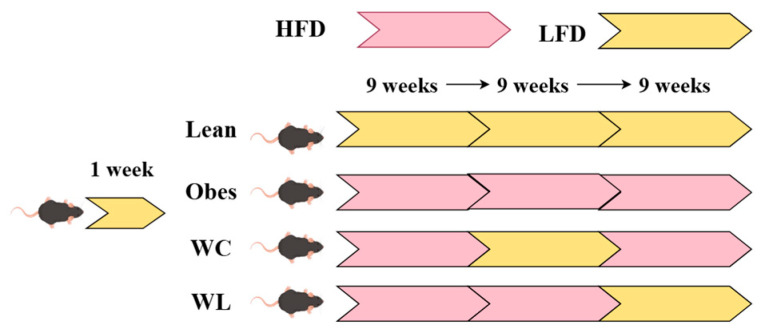
Six- or 7-week-old male C57BL/6J mice were fed LFD for one week to adapt to the environment and then LFD or HFD for indicated blocks of time over 27 weeks. These mice were divided into 4 groups, including a lean group (continuous 27 weeks of LFD feeding), obesity group (continuous 27 weeks of HFD feeding), weight cycling (WC) group (9 weeks of HFD feeding, 9 weeks of LFD feeding and 9 weeks of HFD feeding) weight loss (WC) group (18 weeks of HFD feeding and 9 weeks of LFD feeding). Created using FigDraw (www.figdraw.com, accessed on 20 May 2024).

The weight cycling mice model is an effective experimental method commonly used in clinical analysis of its mechanism. The weight cycling mice model is mainly converted by a high-fat diet and a low-fat diet, causing cyclic changes in body weight, and finally establishing an animal model. At present, there are two main schemes, one is a 9-week high-fat diet, a 9-week low-fat diet plus another 9-week high-fat diet. When constructing this model, male mice of the C57BL/J model about 7 weeks old are usually used. After a week of acclimatization, the mice would divide into different groups at random: the group with obesity (high-fat diet, protein content 16%, carbohydrate content 38%, fat content 46%, total energy 20.5 KJ/g), lean group (conventional diet, protein content 21%, fat content 6%, total energy 15.36 KJ/g), weight cycling group (high-fat diet for 9 weeks, low-fat diet for 9 weeks and high-fat diet for 9 weeks), and weight loss group (high-fat diet for the first 18 weeks and low-fat diet for the last 9 weeks) [45,56,62,75,76] (Figure 2). This is the most common 27-week dietary intervention used to model weight cycling. In addition, there is also a 24-week weight cycling mice model. The process is similar to the 27-week weight cycling model, but the time of each dietary intervention is shortened. In a study of the effect of weight cycling on inflammation, Li et al. [76] randomly divided 30 male C57BL/6J mice into a normal diet group, high-fat diet group, and weight cycling group. The first 8 weeks and the last 8 weeks of the weight cycling group were given a high-fat diet, and the middle 8 weeks were given a normal diet. The normal diet group lasted for 24 weeks, and the obesity group were given a high-fat diet that lasted for 24 weeks. In addition, there was an 80-day weight cycling model. For example, Denkel et al. [16] randomly divided 36 mice into a low-fat diet group, high-fat diet group, and weight cycling group. The low-fat or high-fat diet group was given a low-fat or high-fat diet for 10 weeks. The mice with weight cycling were fed a high-fat diet without restriction for 10 days, and then with a caloric restriction diet for 4 days. The caloric restriction was 70% energy intake during the high-fat diet period. After 4 cycles of this feeding pattern, the weight cycling group was fed with an unlimited high-fat diet for 10 days to ensure body weight recovery, and finally fed with an intensive diet for 2 weeks. The weight and energy intake of all mice were measured at regular intervals during these 80 days.

The animal model of weight cycling can simulate some clinical features and is easy to repeat. Disease characteristics and related mechanisms in overweight or obese populations can be confirmed and explored in animal experiments. However, there are some limitations in that model, such as weight and span not being specified, the laboratory findings being unstable, and some experiment indicators being inconsistent between male and female mice [56]. Therefore, in subsequent studies, it is necessary to clearly define the magnitude of body weight changes during the body weight cycling cycle, as well as to find animal models with more accurate and shorter spans.

## 5. Strategy for Weight Cycling Control

Obesity is a multifactorial disease with an increasing incidence worldwide. Weight loss is easy to achieve, but 80% of individuals who lose weight find it difficult to maintain weight without rebounding. Only about 20% of overweight people seem to be able to successfully maintain weight loss of more than 10% for more than one year [77,78]. There is a need to come up with workable strategies to deal with weight cycling. Exercise training is an important strategy for weight loss and inhibition of weight cycling. Physical activity can effectively reduce the weight of people and promote the reduction of subcutaneous and visceral fat. Exercise has a positive effect on the maintenance of lean body mass during weight loss and subsequent weight maintenance [79,80]. Exercise for weight loss includes aerobic training and resistance training, and the energy consumption caused by the two ways is different. However, no matter what kind of exercise training is carried out, the effect of weight and fat reduction are significantly lower than that of the non-exercise population [80]. In fact, the benefits of exercise on the human body not only reduce weight but also enhance cardiovascular function and increase the content of skeletal muscle mitochondria [79,81]. Exercise improved skeletal muscle mitochondrial content, mitochondrial electron transport chain, and fatty acid oxidase activity in overweight and elderly people with obesity, while increasing β-hydroxyacyl CoA dehydrogenase (β-HAD) and NADH-oxidase activity, which is in stark contrast to the effect of caloric restriction-induced weight loss on mitochondria [81]. At present, the research on exercise control of weight cycling has not been reported. The previously unpublished data of our research group show that exercise can effectively improve weight cycling, and its mechanism may be related to improving the inflammatory state of adipose tissue and improving the health status of obese skeletal muscle.

Low-calorie diets lead to significant weight loss and visceral fat loss [82,83]. Witjaksono et al. [84] studied the effect of a low-calorie high-protein diet and a low-calorie standard protein diet on the waist circumference of visceral obesity in adults with a history of weight cycling. They found that protein composition had no significant effect on waist circumference, but calorie-restricted diet intervention can reduce visceral fat content [84]. Calorie restriction is an effective strategy for weight loss, but weight loss is usually not maintained. Part of the reason is that the body undergoes compensatory physiological adaptation, such as energy consumption, fat oxidation, and appetite-promoting hormone secretion levels to promote weight recovery [85,86]. Studies based on athletes from different sports have observed that sequential weight loss and recovery alters body expression and metabolism. However, compared to non-athletic normal-weight individuals, weight cycling athletes may not have excess fat, and the main reason for this phenomenon is that athletes do not just rely on diets to lose weight [87].

The best strategy for improving weight loss is calorie restriction combined with adequate physical activity [79]; this enhanced mitochondrial electron transport chain capacity and mitochondrial content in obese patients with insulin resistance and type 2 diabetes [88]. Based on multidisciplinary theories, weight loss management plans include caloric restriction, increased physical activity, and behavioral changes. The success of weight loss maintenance is related to behavioral factors (high levels of physical activity, low-calorie and low-fat diets, frequent weight testing), cognitive factors (reduced depression, increased outcome satisfaction, and self-regulation), personal traits, and long-term group help [89]. Enhancing the success rate of weight loss and maintaining the effect of weight loss by improving weight loss strategies is one of the methods to reduce the incidence of weight cycling.

Surgery, drugs, and transgenic methods are also effective strategies to control weight cycling. In the past few decades, bariatric surgery has been proven to improve long-term weight gain and obesity comorbidities. Rounx-in-Y gastric bypass (RYGB) and sleeve gastrectomy (SG) are commonly used in the treatment of obesity, which usually leads to significant weight loss and reduces the risk of some obesity complications [90,91]. In obese women, RYCB reduced body weight independent of changes in food intake and produced significant and sustained weight loss, while having a positive impact on energy homeostasis and the reproductive system [92]. In animal experiments, vertical sleeve gastrectomy (VSG) improved blood glucose control, blood pressure and blood lipid levels in obese mice, and improved their blood glucose homeostasis while losing weight [93]. However, weight gain is a common phenomenon after bariatric surgery. Bariatric surgery also aggravates the degree of illness in morbid patients and even reduces their physical fitness [90]. In terms of drug treatment, the new appetite-regulating weight loss drugs are expected to maintain the weight loss effect. Human adipose tissue can be transformed into thermogenic cells by non-adrenergic stimulation, making adipose tissue a peripheral target for obesity drugs [94]. Some weight loss drugs used in the 19th century, such as thyroid hormones and mitochondrial uncoupler dinitrophenol (DNP), were used to reduce body weight by increasing energy consumption and lipid oxidation. Fenfluramine does not increase the body’s energy expenditure but enhances the thermal effect of food to promote weight loss [95]. Many drug treatments are currently only used in animal experiments and have not been clinically proven. They also have potential side effects and seriously damage cardiovascular and mental health. Some natural plant extracts, such as naringenin and β-carotene, have the potential to safely improve obesity by driving lipolysis and enhancing insulin sensitivity [94]. Gene technology will be the direction of improving obesity treatment in a few years, and some genes that improve obesity and metabolism have been found in animal experiments. Sarcosinemia autosomal recessive (SAR) expression was significantly increased in the adipose tissue of mice with obesity, and the systemic SAR gene knockout reduced diet-induced obesity and improved systemic glucose homeostasis. Angiotensin II type 1a receptor (AT1aR) gene deletion reduced adipocyte hypertrophy and delayed obesity in high-fat diet rats [96,97]. Nicotinamide N-methyltransferase (NNMT) is present in fatty lipids and liver. Knockout of this gene can increase energy consumption and is a new direction for the treatment of obesity and T2DM [98]. Empaglifozin modestly lowers blood pressure and body weight, aside from its cardiac and renal protective properties. In animal experiments, it was observed that empaglifozin prevented HFD-induced metabolic changes and prevented steatosis, which increased energy expenditure and browning of adipose tissue, as well as positively affected the size and morphology of mitochondria in the skeletal muscle of mice fed both a LFD and HFD [99]. Although surgery, drugs, and genetic modification can also play a role in weight control, there are a number of side effects and insecurities to these methods compared with exercise and dieting.

## 6. Conclusions

Weight cycling is a research hotspot in the field of obesity, which increases the risk of various diseases in the body and even exceeds the risk of continuous obesity. However, current research on the mechanisms by which weight cycling affects the body remains at the stage of gene level and specific protein pathways. The studies of weight cycling on pancreas and adipose tissue are deeply microscopic, but it is at the macro level in cardiovascular. Additionally, weight cycling also affects the liver and kidney function. The magnitude and periodicity of weight fluctuations during weight cycling are not standardized. Bariatric surgery, drug therapy, and genetic technology are ways to improve obesity, but these methods have multiple side effects and lack clinical testing. Therefore, scientific exercise combined with a rational diet is one of the safe and long-lasting strategies to improve obesity. In the future, we should also propose strategies to effectively improve physical functioning for patients with weight cycling.

## Data Availability

Not applicable.
